# Development of Photodynamic Antimicrobial Chemotherapy (PACT) for *Clostridium difficile*

**DOI:** 10.1371/journal.pone.0135039

**Published:** 2015-08-27

**Authors:** Luisa De Sordi, M. Adil Butt, Hayley Pye, Darina Kohoutova, Charles A. Mosse, Gokhan Yahioglu, Ioanna Stamati, Mahendra Deonarain, Sinan Battah, Derren Ready, Elaine Allan, Peter Mullany, Laurence B. Lovat

**Affiliations:** 1 Microbial Diseases, UCL Eastman Dental Institute, London, United Kingdom; 2 Research Department of Tissue & Energy, UCL, London, United Kingdom; 3 Division of Gastrointestinal Services, University College Hospital, London, United Kingdom; 4 Department of Chemistry, Imperial College London, London, United Kingdom; 5 PhotoBiotics Ltd, Chemistry Building, Imperial College London, London, United Kingdom; 6 Department of Life Sciences, Imperial College London, London, United Kingdom; 7 Organix Ltd, Colchester, United Kingdom; 8 School of Biological Sciences, University of Essex, Colchester, United Kingdom; 9 Public Health Laboratory London, Pathology & Pharmacy Building, London, United Kingdom; University of Aveiro, PORTUGAL

## Abstract

**Background:**

*Clostridium difficile* is the leading cause of antibiotic-associated diarrhoea and pseudo membranous colitis in the developed world. The aim of this study was to explore whether Photodynamic Antimicrobial Chemotherapy (PACT) could be used as a novel approach to treating *C*. *difficile* infections.

**Methods:**

PACT utilises the ability of light-activated photosensitisers (PS) to produce reactive oxygen species (ROS) such as free radical species and singlet oxygen, which are lethal to cells. We screened thirteen PS against *C*. *difficile* planktonic cells, biofilm and germinating spores *in vitro*, and cytotoxicity of effective compounds was tested on the colorectal adenocarcinoma cell-line HT-29.

**Results:**

Three PS were able to kill 99.9% of bacteria in both aerobic and anaerobic conditions, both in the planktonic state and in a biofilm, after exposure to red laser light (0.2 J/cm^2^) without harming model colon cells. The applicability of PACT to eradicate *C*. *difficile* germinative spores indirectly was also shown, by first inducing germination with the bile salt taurocholate, followed by PACT.

**Conclusion:**

This innovative and simple approach offers the prospect of a new antimicrobial therapy using light to treat *C*. *difficile* infection of the colon.

## Introduction

The emergence of microbial resistance to most of the known classes of antibiotics has led to an urgent need to identify new antimicrobial strategies [1–5].

**Fig 1:**
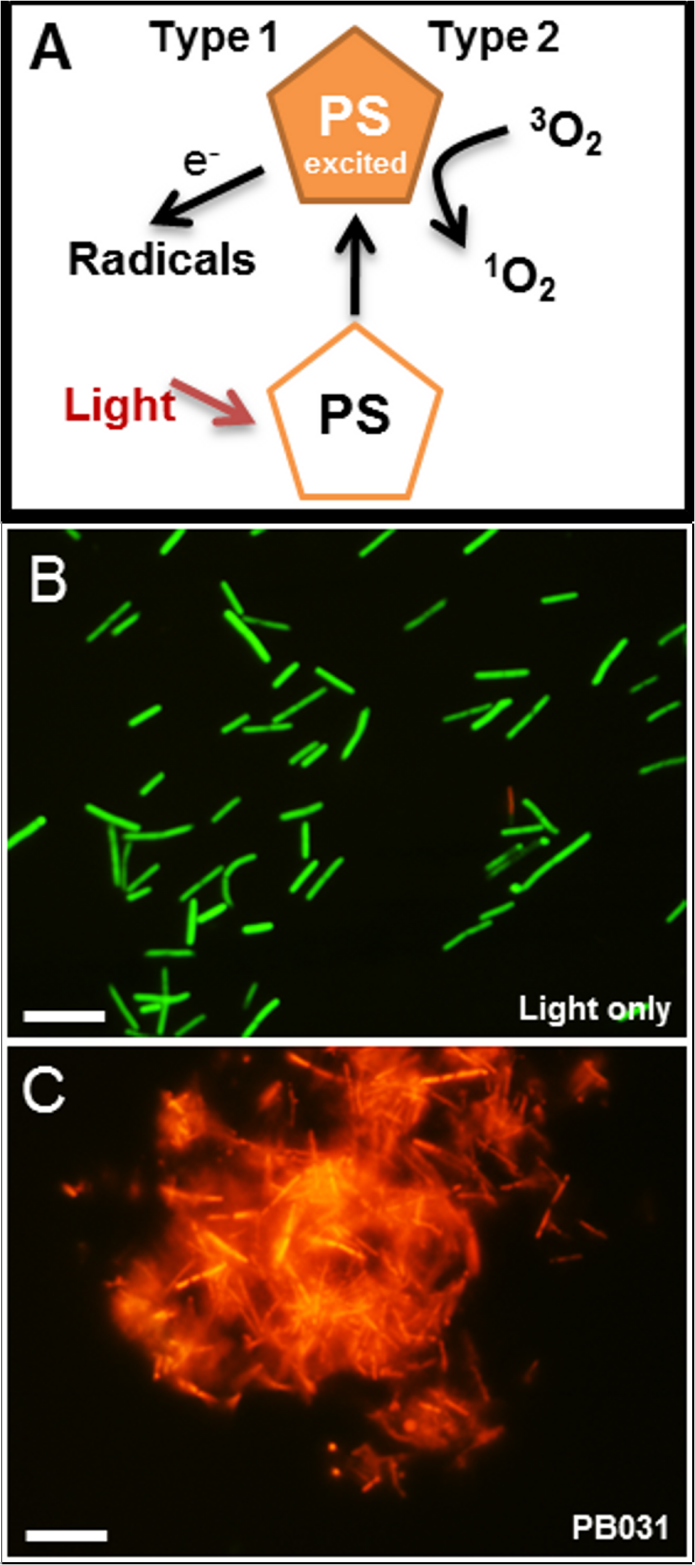
Mechanism of PACT (A) and post PACT live/dead viability assay on *C*. *difficile* with light only (B), and PB031 (C) staining alive (green) and dead (red) bacteria. Scale bars represent 10 μM.

Photodynamic antimicrobial chemotherapy (PACT) involves the combination of a light-sensitive dye, known as a photosensitiser (PS), and locally applied visible light [6,7]. Upon illumination of the PS at one or more wavelengths corresponding to the absorption peaks, the excited molecule can react with a target (molecular oxygen or other targets within biological systems) by electron transfer generating radical species (Type I mechanism). Alternatively, the excitation energy can be transferred from the excited triplet of the PS to triplet dioxygen forming a ground state PS and excited singlet oxygen (Type II mechanism) (Fig 1A). Accumulation of such reactive species, both radical and singlet oxygen, leads to irreversible damage to the target cell.

At present, numerous PS have been developed for the targeting of localised infections and drug-resistant bacteria [8] and no microbial resistance to PACT has been reported so far [9–11]. Despite this, clinical applications are still relatively limited.

The aim of the current work is to evaluate PACT as a treatment for *Clostridium difficile* infections. This Gram positive anaerobic bacterium is the main cause of nosocomial antibiotic‐associated diarrhoea (15%–25% of cases) and is an increasingly common pathogen in the community [12] with a significant economic burden on the healthcare system. *C*. *difficile* infection occurs following disequilibrium in the intestinal microbiota, usually caused by broad-spectrum antimicrobial therapy, or as a result of compromised immunity [13]. Recurrence of symptoms, as a consequence of relapse of the original infection, occurs in 5–47% of cases [14]. The main virulence factors of this organism are the two toxins A and B, however other factors such as sporulation, and antibiotic resistance are also likely to be important[15,16],. Symptoms range in severity from mild to severe with possible serious implications like toxic megacolon (0.4%-3% of the cases) [17–19]. Treatment usually involves the use of vancomycin and metronidazole [17]. Research into new treatments has focused on the development of new antibiotics (i.e. fidaxomicin, tigecycline or nitazoxanide) [17], faecal transplant [20], probiotics [18], monoclonal antibodies against the toxins [21], toxoid vaccines [22,23] or treatment with non-toxigenic strains [24].

The aim of the work presented was to screen and characterise different PS to select the most effective option for the development of PACT against *C*. *difficile* cells, biofilm and germinative spores.

## Materials and Methods

### Strains and growth conditions used in this study

*C*. *difficile* was routinely grown in Brain Heart Infusion (BHI) broth or agar (Oxoid) at 37°C in an anaerobic (10% CO_2_, 10% H_2_, 80% N_2_) workstation (MACS-MG-1000, Don Whitley Scientific). Biofilms were grown in BHI broth supplemented with 0.1% cysteine (BHIS). All experiments were performed on strain R20291 (Anaerobe Reference Laboratory, Cardiff, UK) or CD1457, CD1458, CD1481, CD1490 and CD1523 (Table 1).

**Table 1:** *C*. *difficile* clinical isolates kill following PACT (665nm, 0.24 J/cm^2^) performed with methylene blue, S4, chlorin e6 and talaporfin (100 μM); < 1 log_10_ killing (−); 1 to < 3 log_10_ killing (+); 3 to 4 log_10_ killing (++); > 4 log_10_ killing (+++).

Strain	Ribotype	PS (100 uM)	Light	Dark
CD1457	173	Methylene blue	++	+
		S4	+++	-
		Chlorine e6	+++	+
		Talaporfin	++	-
CD1458	011	Methylene blue	++	+
		S4	+++	-
		Chlorine e6	+++	+
		Talapofin	++	-
CD1481	249	Methylene blue	+++	+
		S4	+++	-
		Chlorine e6	+++	-
		Talaporfin	++	-
CD1490	027	Methylene blue	+++	+
		S4	+++	-
		Chlorine e6	+++	+
		Talaporfin	++	-
CD1523	020	Methylene blue	+++	-
		S4	++	-
		Chlorine e6	+++	-
		Talaporfin	+++	-
R20291	027	Methylene blue	+++	+
		S4	+++	-
		Chlorine e6	+++	-
		Talaporfin	+++	-

### PS used in this study

Methylene blue, talaporfin and chlorin e6 were purchased from Sigma-Aldrich, MedKoo and Frontier Scientific via their UK distributor, Inochem Ltd, respectively. S2 and S4, sulfonated aluminium phthalocyanines, were a generous donation from Professor David Phillips (Imperial College London). PB021, PB031, PB065, PB066, and PB067 consist of various chemical modifications around a central Pyropheophorbide-a (PPa) molecule carried out by chemists led by Dr Gokhan Yahioglu (PhotoBiotics Ltd.) and Dr Ioanna Stamati (Imperial College London) [25]. m-THPC was clinical grade material provided by Quanta Nova. TPC-SNT was donated by Dr Sinan Battah, Organix Ltd. (University of Essex). The compound TPC-SNT is a sulfonated chlorin with beta substituted hydrazon functionality. All structures are shown in S1 Fig.

PB021, PB031, PB065, PB066 and PB067 were all characterised by ^1^H, ^13^C nuclear magnetic resonance (NMR), liquid chromatography–mass spectrometry (LC-MS) and high-resolution mass spectrometry (HRMS) with accurate mass determination and were all ≥ 98% pure. The purity of S2 and S4 were determined to be ≥ 95% by reverse-phase high performance liquid chromatography (HPLC) and characterised by mass spectrometry (MS) (FAB, negative ion mode). mTHPC and TPC-SNT were characterised by NMR, HPLC and electrospray mass spectrometry (ESMS). m-THPC was 98% and TPC-SNT 95% pure. All other PS were obtained from commercial sources and were the best grade available.

Stock solutions of each PS (1 mM) were prepared in sterile phosphate buffer saline (PBS) with the exception of PPa and its derivatives, which were diluted in DMSO. Solutions were kept in the dark at -20°C for a maximum of two weeks before use.

UV-visible absorption spectra of the PS in DMSO or BHI broth were recorded using a Lambda 25 UV/VIS spectrophotometer (Perkin Elmer). Log P and Log D values were predicted computationally with MarvinSketch Freeware Version: 5.7.0 (ChemAxon Ltd. www.chemaxon.com.)

PS were irradiated with the appropriate source of light after irradiance was calibrated using a laser power meter (Gentec TPM-300).

### *In vitro* sensitisation of *C*. *difficile* vegetative cells

*C*. *difficile* was grown with agitation (50 rpm) for 16 hours, typically reaching an optical density (OD)_600_ of 1.0, corresponding to approximately 10^9^ cfu/ml. The cultures were serially diluted in BHI supplemented with the desired concentration of PS or with the PS solvents as control. All dilutions were performed in duplicate and 20 μl of each dilution were spotted onto BHI agar (1 ml contained in the wells of a 24 well tissue culture plate). Plates were set up in duplicate with one plate exposed to light and the other kept in the dark. The plates were protected from light except during the period of exposure to Periowave diode lasers (Ondine Biomedical Inc., Canada). Light delivery was performed within five minutes of PS incubation unless otherwise stated. Plates were returned to the anaerobic cabinet and incubated for 48 hours. Data were collected by recording the presence of colonies. When the experiments were performed in anaerobic conditions, all steps were carried out inside an anaerobic cabinet (MACS-MG-1000, Don Whitley Scientific) and solutions were deaerated in the anaerobic cabinet for a minimum of two hours before use. When PS cell binding or internalisation was to be evaluated, the bacteria were recovered by centrifugation (1070 g, 5 minutes) and washed three times with 1 ml of BHI broth to eliminate extracellular, unbound PS prior to diluting. The effect of PACT with methylene blue, chlorin e6, PB031, S4 and talaporfin on *C*. *difficile* viability was recorded via fluorescent microscopy (Olympus BXS1) using a LIVE/DEAD BacLight Bacterial Viability Kits (Life technologies) according to the manufacturer’s instructions.

### *In vitro* sensitisation of *C*. *difficile* germinative spores

To isolate *C*. *difficile* spores, strain R20291 was grown without shaking in BHI broth for four days and vegetative cells were heat-killed at 65°C for 20 minutes. To determine their sensitivity to PACT, spores were treated with the appropriate PS, with and without co-incubation with 0.1% sodium taurocholate for the desired amount of time, and 20 μl of 10-fold serial dilutions were plated on to BHI agar containing 0.1% sodium taurocholate (1 ml in the wells of a 24 well plate). Plates were returned to the anaerobic cabinet for the appropriate incubation time before laser exposure and incubation as described.

### *In vitro* sensitisation of *C*. *difficile* biofilm

*C*. *difficile* R20291 biofilm was prepared as described by Dawson *et al*. [26] with some modifications. Briefly, 1 ml of pre-reduced BHIS was inoculated with a 1 in 10 dilution of a 16 hour culture of *C*. *difficile* R20291 into each well of a polystyrene 24-well tissue culture plate (BD Falcon). Medium only was added as a control. Biofilms were grown for six days before being washed once with sterile distilled H_2_O (dH_2_O) and covered with 50 μl of a PS solution at the appropriate final concentration. The same amount of dH_2_O was used as a control. Each experiment was performed in the presence and absence of light and each treatment comprised two technical replicates. After treatment, the bacteria were resuspended by vigorous pipetting in 1 ml of BHI broth, washed and serially diluted for viable counts on BHI agar plates which were incubated for 48 hours in anaerobic conditions.

### *In vitro* sensitisation of HT-29 cells

An adherent colorectal adenocarcinoma cell line (HT29) was plated at 30,000 cells per well in black 96-well plates in 200 μl of the cell culture medium DMEM:F12 (Lonza) supplemented with 10% Foetal Calf Serum (FCS) (Invitrogen) and 1% Penicillin-Streptomycin Solution (Sigma-Aldrich). After 24 hours, the medium was replaced with PS solutions in PBS mixed 1:1 with supplemented cell culture medium. Controls comprised cells exposed to cell culture medium:PBS (1:1) (100% viability), medium plus 0.25% Triton X100 (0% cell viability), and medium alone without cells (background absorbance). Every condition was repeated across four different wells (technical replicate n = 4). Plates were protected from light and incubated in a humidified incubator (37°C/5% CO_2_) for 5 minutes or 2 hours. When required, extracellular unbound PS was removed by washing the adherent cells twice with PBS. Cells were then either irradiated at 665nm delivering 0.24 J/cm^2^ over 10 seconds with a Periowave diode lasers or left in the dark. After irradiation, cells were washed once with PBS and then returned to supplemented cell culture medium. After a further 24 hours incubation, the medium was replaced with MTS reagent (Promega) diluted 1:10 into 100 μl un-supplemented cell culture medium and plates were returned to the incubator for 2 hours. Plates were gently shaken for 2 minutes then the absorbance at 490 nm was measured on an ELx800 Absorbance Microplate reader (BioTek).

### Statistical analysis

All experiments were repeated at least three times and consisted of a minimum of two technical replicates. The mean and standard error of the mean (SEM) were calculated and statistical significance was analysed using a two-tailed, unpaired Student T-test.

## Results

### Absorption spectra and characterisation of PS

**Fig 2:**
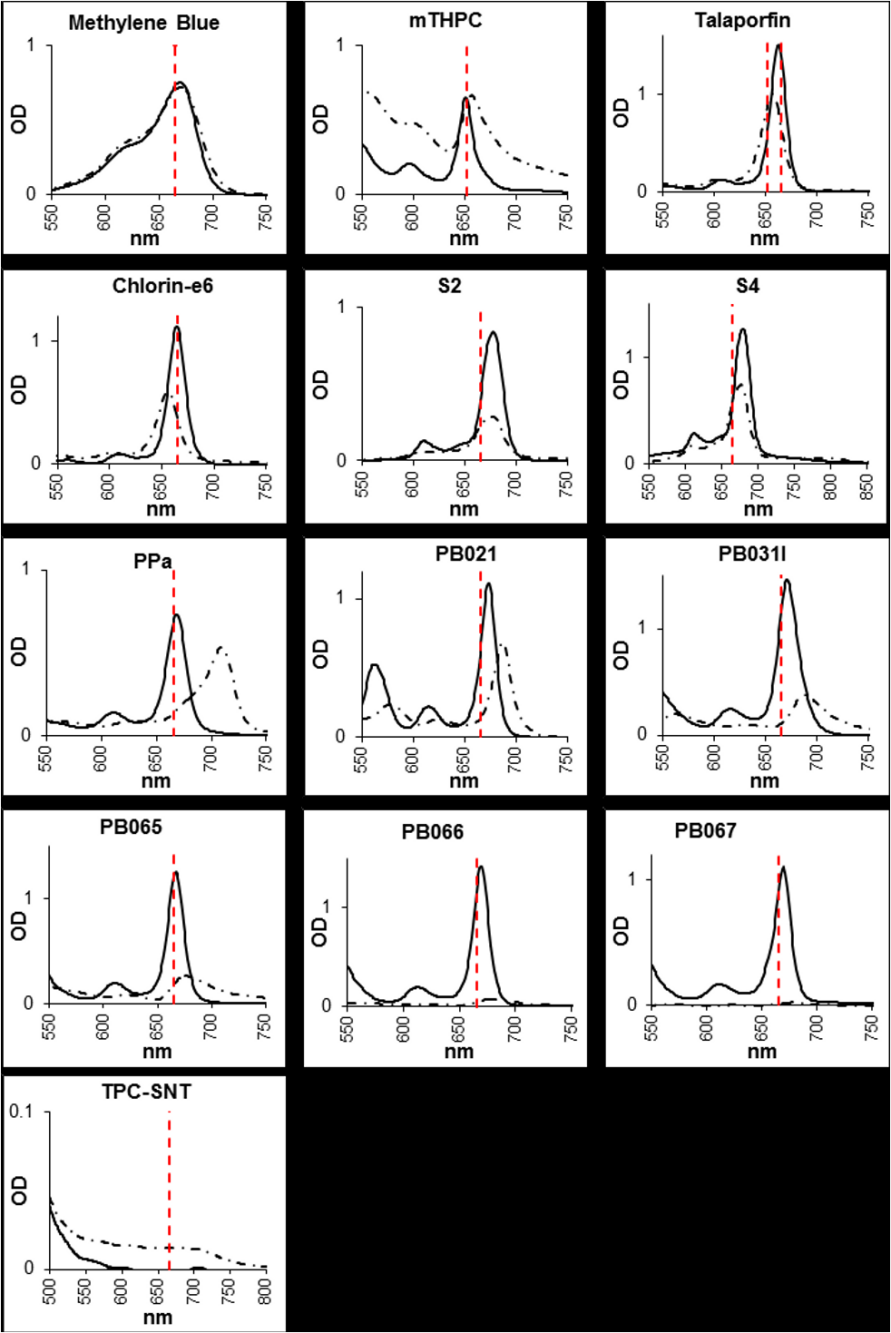
Absorbance spectra of PS in either DMSO (solid line) or BHI (dashed line), overlaid is the relevant laser excitation wavelength in red at 665nm 652nm or 784nm. For spectra comparison, all PS solutions were diluted to 50 μM concentration except Chlorin-e6, PPa and PB067 (25μM), S2 and S4 (6.7 μM), and PB066 (8.35 μM).

Absorption spectra were recorded in different solvents (Fig 2). All PS were soluble in DMSO but not all were fully soluble in BHI. mTHPC in BHI formed a particulate and subsequent scattering of the light was observed in the spectra. If exposed to centrifugal force this came out of solution as a visible pellet. PB031, PB065, PB066 and PB067 all produced significant solid precipitation and the remaining PS were soluble (methylene blue, talaporfin, chlorin e6, S4, S2, PPa, PB021, TPC-SNT).

Absorption peaks (in DMSO and BHI broth) of each PS are shown in Table 2 together with the wavelength of the laser used and Log P and Log D values (indicating hydrophobicity of the compounds in a non-ionised and ionised state respectively). The singlet oxygen quantum yields, where available, were extrapolated from the literature or from the suppliers’ information (Table 2).

**Table 2:** Main characteristics of the PS used in this study.

Photosensitiser (PS)	Abs Max (in DMSO) nm	Abs Max (in BHI) nm	Laser used (nm)	Predicted Log P (Non-ionic species)	Predicted Log D (Ionic species at pH7.4)	Predicted Ionisation at pH7.4	Singlet Oxygen Quantum Yield (Solvent)^Ref^
Methylene Blue	670	670	665	2.61	2.61	1	0.50 (Ethanol)^26^
mTHPC	650	657	665	9.21	9.2	0	0.30 (Ethanol)^26^
Talaporfin	663	656	665 and 652	5.85	-6.55	-4	0.77 (Methanol)^27^
Chlorin-e6	663	656	665	7.02	-2.71	-3	0.61 (Toluene)^26^
S2	678	677	665	7.4	5.13	-1	0.27 (Methanol)^28^
S4	679	677	665	7.01	4.75	-1	0.20 (DMF)^28^
PPa	668	709	665	7.15	3.97	-1	0.50 (Toluene)^29^
PB021	673	687	665	9.34	6.04	-1	0.56 (Toluene)^29^
PB031	671	688	665	3.86	3.86	1	Not available
PB065	669	680	665	8.28	8.28	0	Not available
PB066	668	677	665	7.94	7.94	0	0.14 (Toluene)^30^
PB067	668	none	665	3.84	3.84	1	Not available
TPC-SNT	none	none	665	8.9	-0.61	-4	Not available

### PACT on cultures of *C*. *difficile* in the presence or absence of oxygen

Thirteen PS were tested for their ability to kill *C*. *difficile in vitro*. To date, most PACT mechanisms of action have been shown to require molecular oxygen for bacterial targeting [6]. Therefore, an initial screening was performed in which light was delivered in the presence of oxygen. *C*. *difficile* killing was compared to an untreated control kept in the dark. Cells were treated with 14 different PS at concentrations of 10 and 100 μM. Red or Near Infrared (NIR) laser light was delivered at either 665, 652 or 784 nm and was matched to each PS depending on their absorbance profile. Light was delivered at a relatively low dose for 10 seconds with light dose between 0.24 and 0.71 J/cm^2^.

Red or NIR light alone did not show any reduction in viable bacteria. The results of *C*. *difficile* killing experiments are shown in Table 3. Methylene blue was the only PS to kill in the dark (1 log_10_ reduction in bacterial numbers) but its effect was amplified by the delivery of red light (> 4 log_10_ reduction in bacterial numbers) (Table 3). Bacterial kill was also visualised by BacLight Live/Dead assay (Fig 1B and 1C).

**Table 3:** PACT induced *C*. *difficile* kill (strain R20291) in aerobic (+O_2_) and anaerobic (-O_2_) conditions with light, no PS (L^+^ PS ^-^); no light, no PS (L^-^ PS ^-^); light and PS (L^+^ PS ^+^); no light and PS (L^-^ PS ^+^). < 1 log_10_ killing (−); 1 to < 3 log_10_ killing (+); 3 to 4 log_10_ killing (++); > 4 log_10_ killing (+++).

PS		L^+^ PS ^-^	L^-^ PS ^-^	L^-^ PS ^+^	L^+^ PS ^+^ (+O_2_)	L^+^ PS ^+^ (-O_2_)	Energy (J/cm^2^)
Methylene blue	100	-	-	+	+++	+	0.24
	10	-	-	+	++		0.24
mTHPC	100	-	-	-	-		0.24
	10	-	-	-	-		0.24
Talaporfin	100	-	-	-	+++	+++	1.4
	10	-	-	-	+		0.24
Chlorin e6	100	-	-	-	+++	+++	0.24
	10	-	-	-	+++		0.24
S2	100	-	-	-	++	+	0.24
	10	-	-	-	-		0.24
S4	100	-	-	-	+++	+++	0.24
	10	-	-	-	+++		0.24
PPa	100	-	-	-	+++	-	0.24
	10	-	-	-	+++		0.24
PB021	100	-	-	-	-		0.24
	10	-	-	-	-		0.24
PB031	100	-	-	-	+++	+++	0.24
	10	-	-	-	+++		0.24
PB065	100	-	-	-	+++	-	0.24
	10	-	-	-	+++		0.24
PB066	100	-	-	-	+++	-	0.24
	10	-	-	-	+++		0.24
PB067	100	-	-	-	+		0.24
	10	-	-	-	-		0.24
TPC-SNT	100	-	-	-	-		0.24
	10	-	-	-	-		0.24

PS showing a significant bactericidal effect, defined as ≥3-log_10_-unit killing (99.9%) of the initial inoculum (Clinical and Laboratory Standards Institute 1999) ^25, 26^ were selected for further characterisation and the working concentration of 100 μM was used throughout the study to maximise the chance of future *in vivo* efficacy. The antimicrobial activity of the nine PS (methylene blue, talaporfin, chlorin e6, S2, S4, PPa, PB031, PB065 and PB066) (Table 3) fulfilling these criteria were also tested in the absence of oxygen to mimic the conditions found in the colon, and four PS maintained their activity in these conditions (Table 3).

The four PS that were able to kill *C*. *difficile* in anaerobic conditions (talaporfin, S4, chlorin e6 and PB031) were selected for further characterisation together with methylene blue, since it is already approved for use in humans[27–29].

### Blue light killing of *C*. *difficile* in the absence of PS

A 410 nm LED light delivery system (Enfis High-Power LED light engine) was also tested. Many of the PS tested also had absorbance peaks in the blue range of the spectra and their bactericidal activity was tested in conjunction with these wavelengths. However, blue light alone in aerobic conditions was sufficient to kill *C*. *difficile* in the absence of PS. Different light doses were administered: 0.24 J/cm^2^ (0.018 W/cm2 for 13 seconds) resulted in a greater than 3 log_10_ reduction in bacterial numbers and 0.54 J/cm^2^ (0.018 W/cm2 for 30 seconds) resulted in a 4 log_10_ reduction (S2 Fig). Interestingly, these wavelengths did not show any antimicrobial activity against *Enterococcus faecium*, *Pseudomonas aeruginosa* or *Escherichia coli* excluding a general antimicrobial mechanism (data not shown). Exposure of *C*. *difficile* to blue light in anaerobic conditions did not cause any reduction in bacterial numbers (data not shown) and delivery of blue light did not affect the viability of *C*. *difficile* spores (data not shown).

### PS binding and internalisation in the target cell

PS (100 μM) were incubated with bacteria for 5 minutes or 2 hours before being removed by washing with BHI broth prior to PACT. The level of bacterial killing after light delivery (665 nm, 0.24 J/cm^2^) was then compared with that of the unwashed bacteria. Table 4 shows that chlorin e6 and PB031 retained full antimicrobial activity when incubated with the bacteria for only five minutes prior to washing and light exposure. This suggests that these PS are rapidly taken into the bacterial cytoplasm or are bound within the cell wall or to the cell surface. In contrast, S4, methylene blue and talaporfin showed no antimicrobial activity even when incubated with the bacteria for two hours prior to washing and light exposure, suggesting that they are active from an extracellular location.

**Table 4:** The effect of cell washing on PACT-mediated killing of *C*. *difficile* (strain R20291) by different PS; < 1 log_10_ killing (-); > 4 log_10_ killing (+++)

PS	Kill	Kill	Kill
	5 min incubation	5 min incubation	2 hours incubation
	no wash	+ wash	+ wash
Methylene blue	+++	-	-
Talaporfin	+++	-	-
Chlorin e6	+++	+++	+++
S4	+++	-	-
PB031	+++	+++	+++

### Cytotoxicity of PS to HT-29 colorectal cells

**Fig 3:**
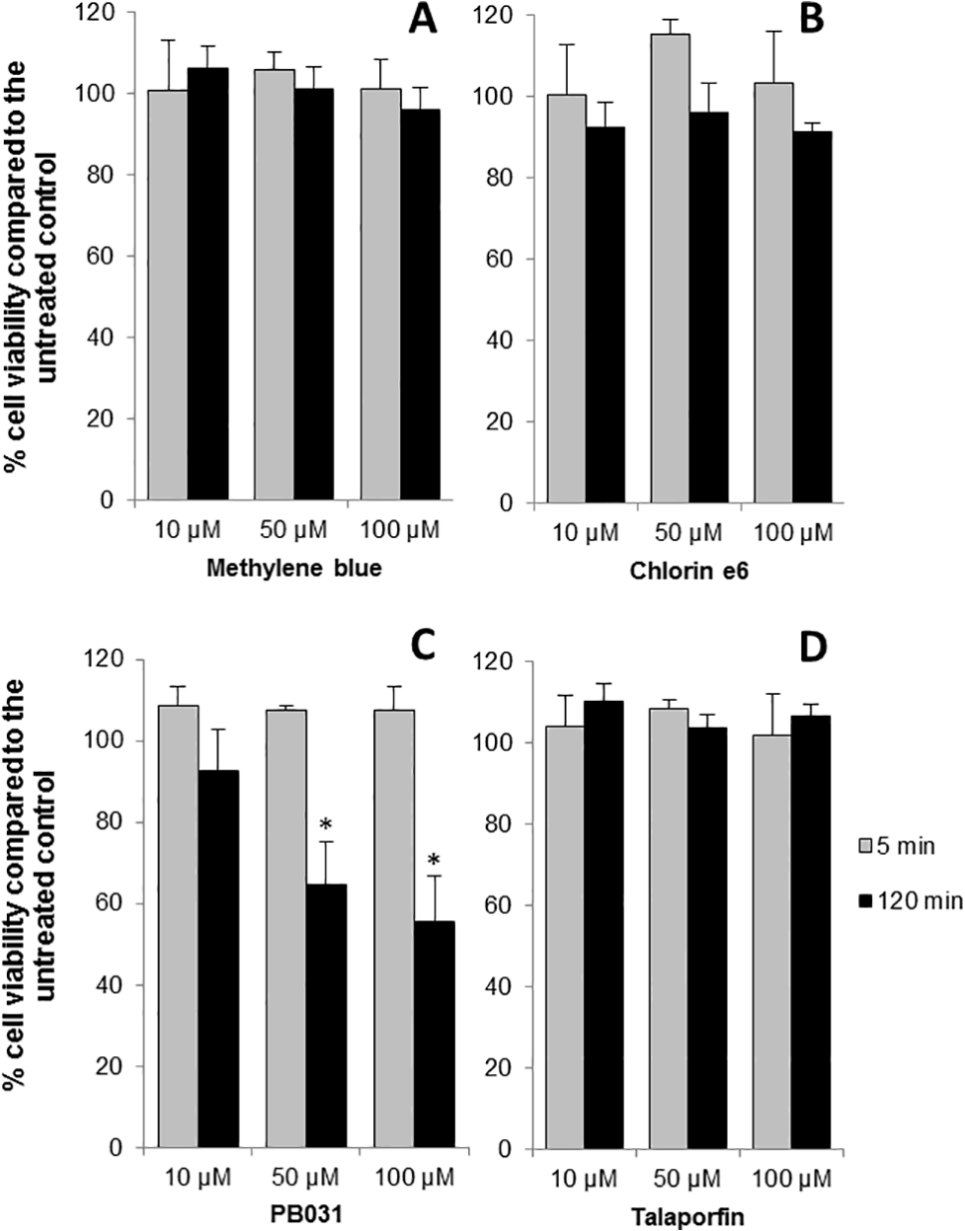
Colon cell survival. Percentage cell viability of cultured HT-29 cells 24 hours after a 5 or 120 minutes incubation with four photosensitiser (PS) and light delivery (665nm, 0.24 J/cm^2^) compared to the untreated control kept in the dark. Bars represent the mean of three biological repeats with error bars indicating SEM. * p < .05.

Cytotoxicity of PS (methylene blue, chlorin e6, PB031 and talaporfin) to mammalian cells was evaluated in the colorectal adenocarcinoma cell line, HT-29. PS cytotoxicity was determined at concentrations of 100 μM, 50 μM and 10 μM and red light was delivered for 10 seconds using a laser emitting at 665 nm with energy of 0.24 J/cm^2^ (0.024W/cm2 for 10 seconds). The PS were incubated with the HT-29 cells for 5 minutes or 2 hours and then irradiated. After 5 minutes, none of the PS showed any cytotoxicity to the colonic cells, whereas after 2 hours, PB031 exhibited a cytotoxic effect at 50 μM and above (Fig 3) and was therefore not characterised further. PS removal by washing prior to laser exposure did not reduce the toxic effect of PB031 at these concentrations (data not shown). S4 was also found to be non-cytotoxic but the experiment was performed once with four technical repeats due to a limited supply of the PS (data not shown). Light or PS alone did not impact on cell viability (data not shown).

In order to assess the maximum dose of light that could be administered without PS cytotoxicity, the light power was increased to up to 7.2 J/cm^2^ (0.024 W/cm2 for up to five minutes) with a PS concentration of 50 μM. The results showed that methylene blue and talaporfin remained non-toxic to HT29 cells whereas chlorin e6 became significantly cytotoxic at a light power of 1.44 J/cm^2^ and S4 was toxic at 7.2 J/cm^2^ (S3 Fig).

### PACT on *C*. *difficile* clinical isolates belonging to different ribotypes are equally susceptible to PACT

Five recent *C*. *difficile* clinical isolates belonging to different ribotypes were tested for susceptibility to PACT using PS that were active against *C*. *difficile* R20291 but not cytotoxic to HT29 cells (methylene blue, S4, chlorin e6 and talaporfin) (Table 1). All the PS showed significant bactericidal activity under the same light conditions that killed strain R20291, causing > 3 log_10_ reduction in bacterial numbers. Methylene blue and chlorin e6 showed limited bactericidal activity in the dark with three of the five strains (Table 1).

### PACT treatment of *C*. *difficile* germinative spores

**Fig 4:**
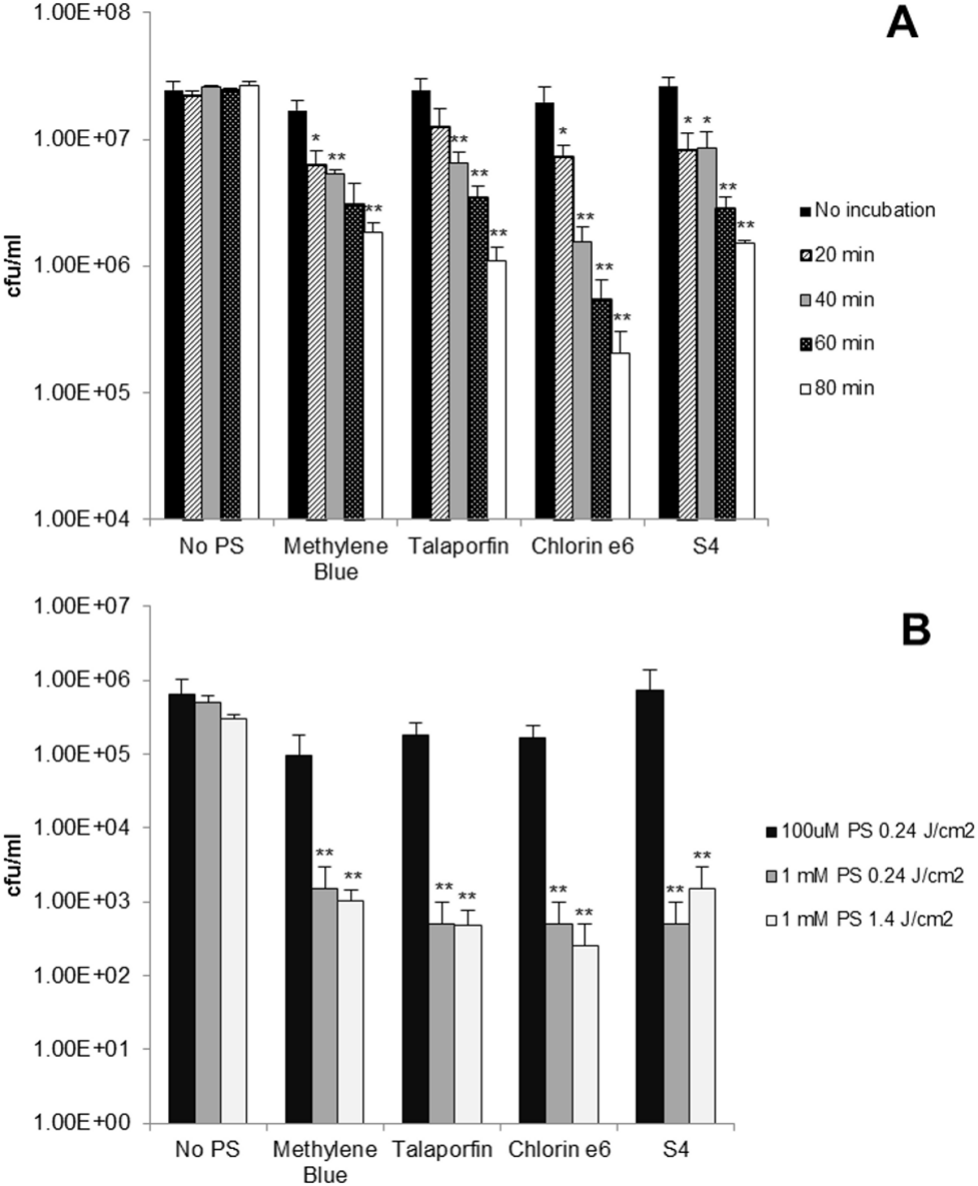
(A) PACT on *C*. *difficile* spores; viable count of germinated *C*. *difficile* post PACT (100 μM PS, 665nm,) after different incubation times with 0.1% taurocholate. (B) Number of bacteria within a *C*. *difficile* biofilm after PACT (100 μM or 1mM PS, 665nm, 0.24 or 1.4 J/cm^2^). Bars represent the mean of three biological repeats and the error bars indicate SEM, * p < .05, ** p < .001.

PACT was assessed for its ability to kill *C*. *difficile* spores *in vitro*. At concentrations of 100 μM and light delivered at 0.24 J/cm^2^, none of the PS was able to reduce the numbers of *C*. *difficile* spores. Increasing the concentration of methylene blue to 5 mM and the laser energy to 14.4 J/cm^2^ in 10 minutes also failed to reduce the numbers of *C*. *difficile* spores (data not shown). Sodium taurocholate was used to induce germination of *C*. *difficile* spores prior to PACT. Sodium taurocholate is a primary bile salt present in the biliary tract and intestines of humans [30]. Treatment with this salt has been shown to be safe in humans[31–33]. A concentration of 0.1% sodium taurocholate was used as it induced the highest rate of spore germination compared to other concentrations (0.001% 0.01% and 1%) (data not shown). Incubation of spores in the presence of 0.1% germinant and 100 μM PS for a minimum of 20 to 40 minutes followed by treatment with red light (665 nm, 0.24 J/cm^2^ in 10 seconds) resulted in significant reductions in the numbers of *C*. *difficile* germinative spores (Fig 4A). Incubation of the spores for the same amount of time with PS alone did not result in any reduction in spore numbers (data not shown). Any combinational cytotoxity of PS and sodium taurocholate (0.01%, 0.1% and 1%) was excluded by testing in HT-29 cells (data not shown).

### PACT on *C*. *difficile* biofilm in vitro

A six-day-old biofilm of strain R20291 was exposed to the four PS; methylene blue, S4, chlorin e6 and talaporfin, and irradiated. Cell adherence was quantified by crystal violet staining before PACT, and was equal to an OD_595_ of 1.04 (± 0.15). Irradiation of the biofilm for 10 seconds (energy delivered 0.24 J/cm^2^ in 10 seconds) showed that the biofilm was resistant to 100 μM PS but susceptible to 1 mM, with a 2 to 3 log_10_ reduction in bacterial numbers (Fig 4B). Further exposure to the laser for one minute using 1 mM PS did not show any further increase in bacterial kill (Fig 4B). No reduction in bacterial numbers was apparent when the biofilm was exposed to PS in the dark (data not shown).

## Discussion

This work is the first study to assess the efficacy of PACT to treat *C*. *difficile* infections. Previous work on the photodynamic targeting of this organism focused on the decontamination of hospital surfaces with the PS, toluidine blue and rose bengal [34] or blue light therapy [35], with the latter showing activity against bacterial spores as well as vegetative cells.

In our work, nine PS out the 14 tested caused a reduction in bacterial numbers of more than four log_10_ and four (chlorin e6, talaporfin, S4 and PB031) showed antimicrobial activity under anaerobic conditions, making them particularly suitable for treatment of *C*. *difficile* within the human colon. Therefore, these PS were investigated further alongside methylene blue, since this molecule is already approved for clinical use [29] and could be more rapidly taken forward for pilot clinical studies. Similarly, since 2004 talaporfin has been approved in Japan for lung cancer treatment [36] and is currently in phase III clinical trials in the USA [37].

Although the exact mechanism of action of the PS is yet to be determined, we propose that methylene blue, S4, and talaporfin might be bactericidal via an extra-cytoplasmic target since washing of these PS resulted in loss of bactericidal activity. On the other hand, chlorin e6 and PB031 retained their ability to kill *C*. *difficile* after washing, suggesting that these molecules are taken up by the bacteria or are tightly bound within the cell wall or at the cell surface from where they inflict damage. Similar conclusions were reached by Huang *et al*. [38] who demonstrated that washed *Staphylococcus aureus* cells incubated with methylene blue survived after light irradiation, whereas a conjugate between polyethylenimine and chlorin(e6) (PEI-ce6) was still active after washing.

In our study, we have shown that internalisation of the PS is not necessary for PACT in *C*. *difficile*. The photodynamic inactivation of both Gram-positive and Gram-negative bacteria via the generation of extracellular ROS have been previously described [39,40] and shown to be dependent on a sufficient PS per cell ratio [41]. However, cell surface binding or internalisation is expected to be advantageous in the gut environment as it will result in higher PS concentrations at the site of infection at the time of light delivery.

A major parameter contributing to successful PACT is the optimal excitation of PS with the correct light wavelength. It should be noted that for some PS, the excitation wavelength did not match exactly with their absorption peaks, and that some PS exhibited shifts in absorption spectra upon dissolution in DMSO or BHI.

PB021, mTHPC, PB067 and TPC-SNT did not kill *C*. *difficile* significantly (less than 3 log_10_ kill) in BHI medium. The inactivity of TPC-SNT is explained by the fact that its absorption spectra did not precisely correspond with the lasers used. The absorption spectra of PB021 in DMSO corresponded with the excitation laser light but once dissolved in BHI, a shift away from the laser wavelength is observed (see Fig 2). The parent molecule (PPa) also showed a similar shift in absorbance but still had significant bactericidal effect. Examination of the structure of PB021 suggests it may be more capable of forming multimeric micelle structures via its hydrophobic centre and hydrophilic PEG-like tail; this is supported by a prolonged retention time in size exclusion chromatography (data not shown). As well as spectral shifts reducing laser light absorption, a change in absorption spectra of a PS indicates that the energy levels available for excitation and/or transition back to ground state could be altered and this can directly compromise PACT. The inactivity of PB067 can be explained by its complete solid precipitation out of BHI solution producing negligible absorption spectra at any wavelength, and the inactivity of mTHPC could be related to its production of a turbid suspension solution upon dissolution in BHI, both these characteristics are reflected in their absorption spectra (see Fig 2).

PB031, PB065, and PB066 are all related compounds and all showed significant bactericidal effect. PB031, a version of this family of compounds with a constant positive charge, was shown to be internalised by the bacteria or bound to the bacterial surface. All three PS also showed some (but not complete) solid precipitation in BHI broth suggesting that in the test environment, sufficient amounts of the monomeric PS were present to achieve a bactericidal result or the bacterial cells counteracted any solvation effects seen in BHI alone.

Five PS (talaporfin, methylene blue, chlorin e6, S2 and S4) were soluble in BHI and retained their spectral characteristics better between solvents. These PS all showed 3 log_10_ oxygen-dependent bacterial kill, and all showed some oxygen-independent bacterial kill. Talaporfin, chlorin e6, and S4 maintained 3 log_10_ kill in anaerobic conditions. In an anaerobic environment, type I PACT mechanisms will dominate so PS that show significant bacterial kill in both anaerobic and aerobic conditions are likely type I dominant and PS that show a drop in bacterial kill in anaerobic conditions are likely Type II dominant. Although a single predominant mechanism for PACT has been inferred for each PS, these mechanisms may in fact differ for the same PS when evaluated under different environmental conditions [42,43]. Further photophysical or molecular experimentation would have to be carried out to determine the exact reason for differences in PS suitability for *C*. *difficile* PACT.

Our leading PS were shown to be equally effective towards five additional recent *C*. *difficile* clinical isolates of different ribotypes indicating that their efficacy is not limited to particular genotypes.

Microorganisms are known to be able to colonise their host, forming a tri-dimensional matrix composed of adherent bacteria embedded in exo-polymeric substances (EPS), known as a biofilm. This structure can protect microbial cells from environmental stress and therapeutic agents including PACT [44]. An *in vitro C*. *difficile* biofilm was recently described [26,45] and the data presented here shows that a biofilm displays higher resistance to PACT compared to planktonic cells. A 10-fold increase in the PS concentration (1mM) compared to that used against planktonic cells, reduced *C*. *difficile* viability by approximately 2.5 to 3 log_10_. The need for higher PS concentrations might be due to limited diffusion in the EPS matrix [46] and the fact that a further increase in light energy did not increase the PS antibacterial effect suggests that either a small portion of cells always remains protected when embedded in the matrix, or that PS photobleaching may occur. Additionally, PACT-resistant spores are likely to be present within the biofilm which can germinate after treatment. Repeated PACT or the addition of biofilm disrupting agents would be required if the biofilm mode of growth is relevant to *C*. *difficile* within the colon, together with the co-delivery of germinants.

Although some PS were previously shown to inactivate *Bacillus* spores [47–49] PACT was not successful in reducing the number of *C*. *difficile* spores under the conditions shown to kill vegetative cells, confirming their high resistance [50], and the induction of germination was required to effect killing. This indicates that future studies aimed at the eradication of *C*. *difficile* spores from patients and/or contaminated surfaces should include taurocholate, its functional groups or analogous molecules [51] to induce germination prior to PACT. Maclean *et al*. [35] previously showed up to 4 log_10_ reduction in the numbers of *Bacillus* spp. and *C*. *difficile* spores upon illumination with 405 nm blue light at a much higher dose of 1.73 kJ/cm^2^, making it a promising method for environmental decontamination but not for the treatment of disease.

The killing of *C*. *difficile* planktonic cells with blue light was also shown [35], but our studies have now revealed that this is achievable at much lower light energies. Interestingly, blue light has antimicrobial activity against a number of bacteria [52] by targeting endogenous metabolites that can act as internal PS [53–55] and *C*. *difficile* is known to synthesise a number of porphyrin compounds which typically absorb in the blue region of the spectra. In agreement with the work of Maclean *et al*. [56] on *S*. *aureus*, this mechanism is oxygen-dependent, and no killing was observed in anaerobic conditions (data not shown). Although blue light was non-toxic to the HT-29 cell line (data not shown), its utility in treating *C*. *difficile* is questionable *in vivo* due to the predicted lower penetration depth in the colonic folds compared to red light [57].

The cancer cell line HT-29 was selected for evaluation as it is derived from human colorectal epithelium, the microenvironment upon which *C*. *difficile* reside in humans from which they cause pathogenicity. It is known that tumour cells are more sensitive than normal cells to the effect of PS. Oseroff *et al*. [58] evaluated a panel of rhodamine and cyanine dyes *in vitro* and found the most effective PS caused marked toxicity to human squamous, bladder and colon carcinoma cells lines but was minimally toxic normal human keratinocytes or monkey kidney epithelial cells under the same conditions. PS are also retained for longer periods in tumour than normal cells *in vivo*, achieving selectivity and increased sensitivity via this mechanism [59]. If PS evaluated were not toxic to HT-29 cells, it is inferred that it is unlikely that they would be toxic to normal colorectal epithelium under the same conditions. Our cell toxicity experiments showed that the PACT conditions that kill *C*. *difficile in vitro* do not affect the viability of colonic cells *in vitro*, with the exception of PB031.

In summary, this work showed potential for PACT as a treatment for *C*. *difficile* induced disease. Non-cytotoxic PS showed significant bactericidal activity against *C*. *difficile in vitro* in both aerobic and anaerobic conditions making them good candidates for *in vivo* studies. The characterised PS showed that antimicrobial activity can occur when PS are either internalised/bound to the cell or located extracellularly, suggesting that specific targeting of the PS for example, using antibodies or bacteriophage, might increase PACT efficiency further. Finally, co-delivery of spore germinants allowed killing of *C*. *difficile* spores suggesting future strategies of fighting recurrence of infections using natural or synthetic germinants in combination with antimicrobial therapy.

## Supporting Information

S1 Fig Structures of the PS used. (TIF)

S2 Fig *C*. *difficile* killing by blue light. Bacteria were quantified as cfu/ml 48 hours after the treatment. Bars represent the mean of four biological repeats and the error bars indicate SEM. The limit of detection was 10^5^ cfu/ml.
(TIF)

S3 Fig Percentage HT-29 cell viability following treatment with PS (50 μM) and red laser light at different energy doses compared to the untreated control kept in the dark. Bars represent the mean of three biological repeats and the error bars indicate SEM, * p < .05.
(TIF)
