# Supplementary figures and images for: Development of Photodynamic Antimicrobial Chemotherapy (PACT) for Clostridium difficile
Source: PLoS One. 2015 Aug 27;10(8):e0135039. doi: 10.1371/journal.pone.0135039 (PMC4551672; doi:10.1371/journal.pone.0135039)

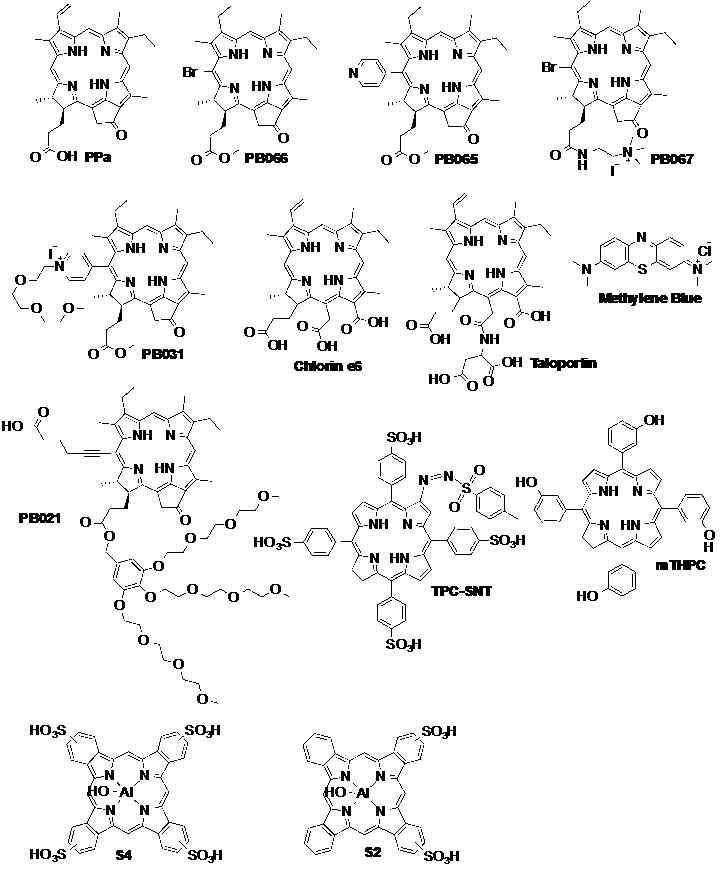

Supplement: S1 Fig — (TIF) [file pone.0135039.s001.tif]

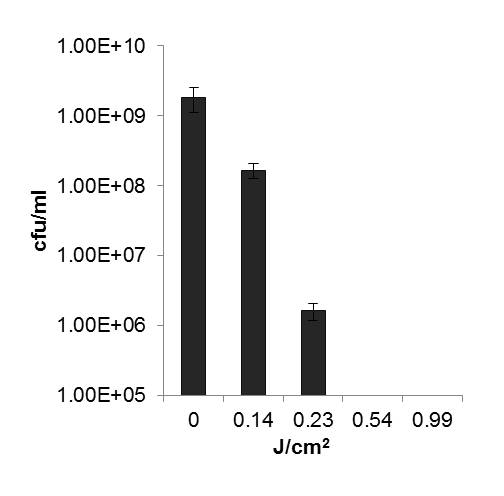

Supplement: S2 Fig — Bacteria were quantified as cfu/ml 48 hours after the treatment. Bars represent the mean of four biological repeats and the error bars indicate SEM. The limit of detection was 105 cfu/ml. (TIF) [file pone.0135039.s002.tif]

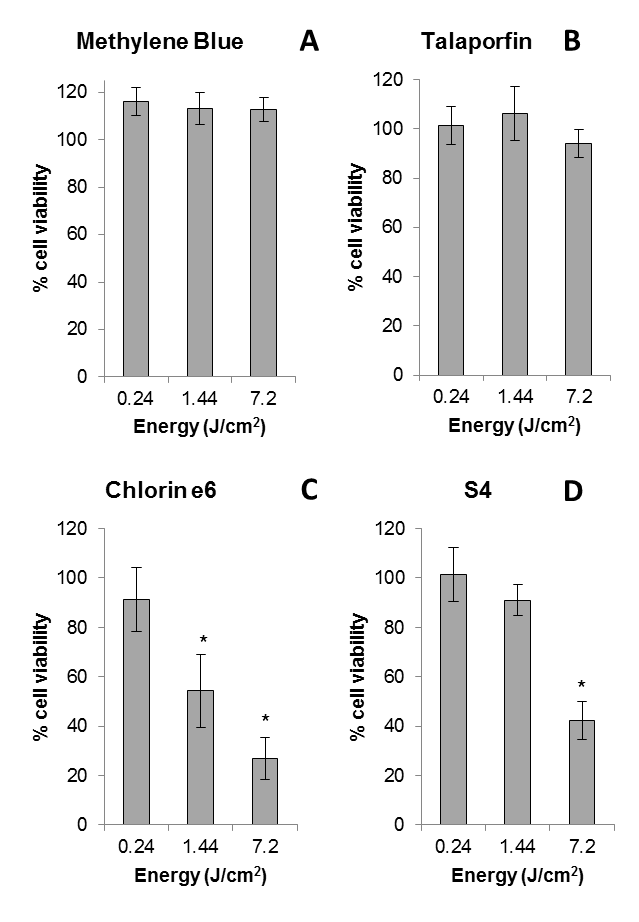

Supplement: S3 Fig — Bars represent the mean of three biological repeats and the error bars indicate SEM, * p < .05. (TIF) [file pone.0135039.s003.tif]
